# *Streptococcus gordonii* infective endocarditis complicated by brain abscess in a patient with a congenital bicuspid aortic valve: a case report

**DOI:** 10.1093/ehjcr/ytad590

**Published:** 2023-11-27

**Authors:** Alysha Bhatti, Viral Sagar, Katie McFaul

**Affiliations:** Department of Infectious Diseases, St George’s Hospital, Blackshaw Road, Tooting, London SW17 0QT, UK; Department of Cardiology, St George’s Hospital, Blackshaw Road, Tooting, London SW17 0QT, UK; Department of Infection and Acute Medicine, St George’s Hospital, Blackshaw Road, Tooting, London SW17 0QT, UK

**Keywords:** *Streptococcus gordonii*, Infective endocarditis, Bicuspid aortic valve, Brain abscess, Case report

## Abstract

**Background:**

Infective endocarditis is associated with significant morbidity and mortality. Oral trauma through dental procedures can result in infective endocarditis through displacement of commensal organisms into the bloodstream. *Streptococcus gordonii* is an oral commensal and is rarely implicated as a cause of infective endocarditis but should be considered in febrile patients with a recent history of odontological procedures.

**Case summary:**

We present a case of a previously healthy 26-year-old woman who presented with a 2-month history of fevers. Blood cultures on admission were positive for *S. gordonii*. Echocardiography demonstrated a congenital bicuspid aortic valve with vegetations and abscess, supporting a diagnosis of infective endocarditis. A magnetic resonance imaging (MRI) brain revealed a small cerebral empyema. She was treated with intravenous antibiotics and underwent an aortic valve replacement.

**Discussion:**

Bicuspid aortic valve predisposes to infective endocarditis, and these patients have higher incidence of requiring cardiac surgery*. Streptococcus gordonii* belongs to the viridans group streptococci that are recognized as causative organisms for infective endocarditis particularly where dental sources are suspected. Patients with infective endocarditis may develop neurological sequelae including cerebrovascular accidents or central nervous system infections. If risk of haemorrhagic transformation is low, surgical intervention for valve replacement should not be delayed.

**Learning points**

- *Streptococcus gordonii* is a potentially rare causative organism of infective endocarditis.
- Aortic valve endocarditis complicated by peri-valvular abscess confers a worse prognosis and necessitates early surgical intervention.
- Silent septic cerebral emboli have low rates of haemorrhagic transformation and should not delay cardiac surgery.
- Antibiotic prophylaxis for infective endocarditis is limited to high-risk patients undergoing at-risk dental procedures whilst oral and cutaneous hygiene is universally recommended.

## Introduction

*Streptococcus gordonii*, an oral commensal and member of the viridans group streptococci (VGS), is an infrequent cause of infective endocarditis. In those with a bicuspid aortic valve (BAV), however, a microbiological preponderance towards VGS has been observed.

Infective endocarditis carries an inpatient mortality of around 20%, conferring a worse prognosis if complicated by peri-aortic abscess and septic emboli. Early identification and surgical intervention are paramount.^1^

We describe a healthy patient with BAV endocarditis secondary to *S. gordonii* who developed septic and neurological complications.

## Summary figure

**Figure:**
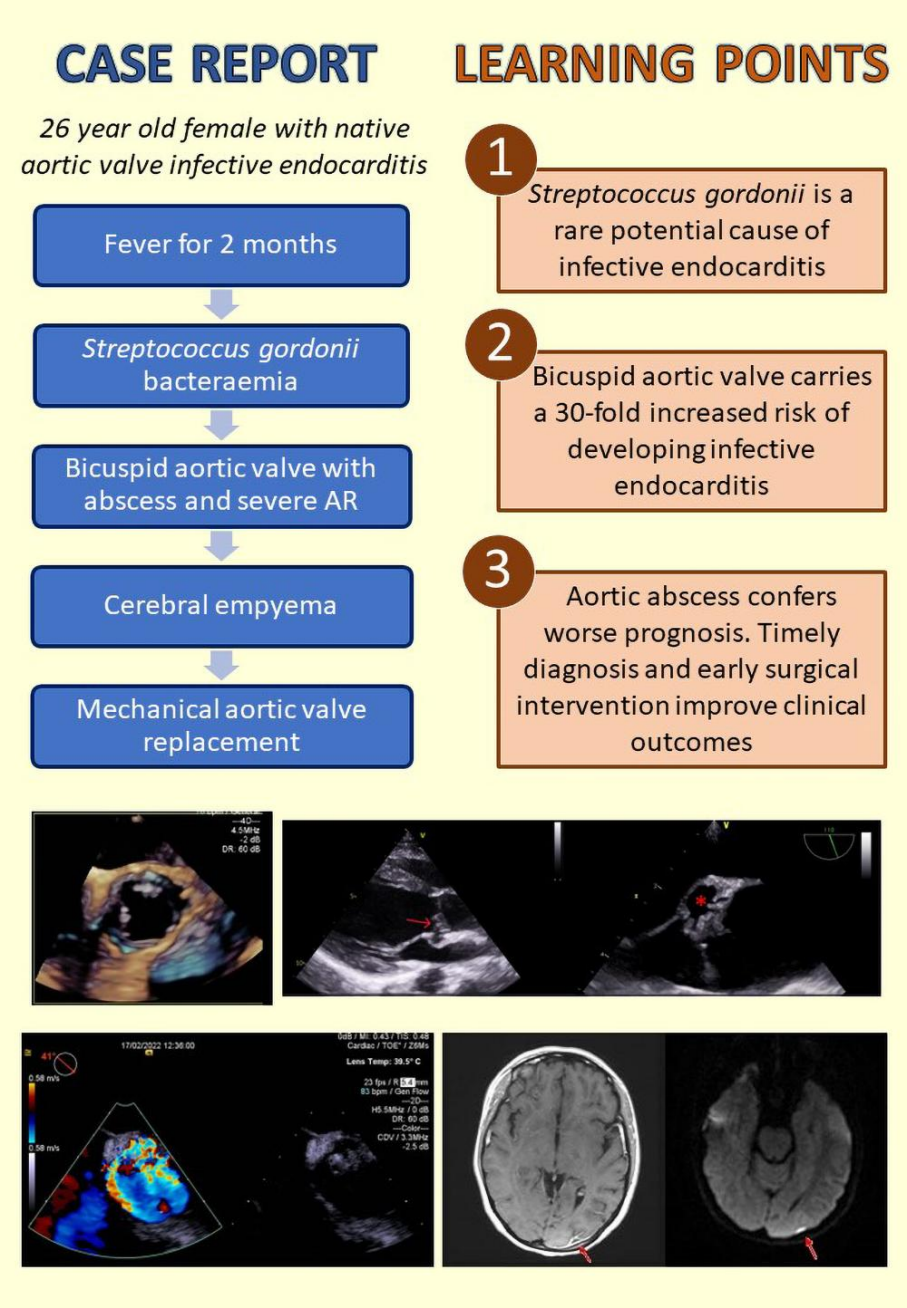


## Case presentation

A 26-year-old female with no past medical history was referred to acute care by her primary care provider with fever and haematuria. She described 2 months of fevers up to 40°C, malaise and night sweats. Her primary care provider diagnosed a urinary tract infection due to a positive urine dipstick for blood and she received two courses of antibiotics that failed to improve symptoms. At presentation, she reported a dental scaling procedure 1 month after the onset of fevers. Blood cultures were obtained, and she was discharged to the community. Within 48 h, these grew *S. gordonii*, and she was recalled by the clinical infection unit.

On examination, her respiratory rate was 16 breaths/min, SpO_2_ 99%, blood pressure 116/74 mmHg, pulse 85 b.p.m., and temperature of 36.7°C. She had an early diastolic murmur loudest in expiration at the left lower sternal edge. There were no peripheral stigmata of infective endocarditis or signs of dental infection and no features of cardiac failure.

**Figure 1:**
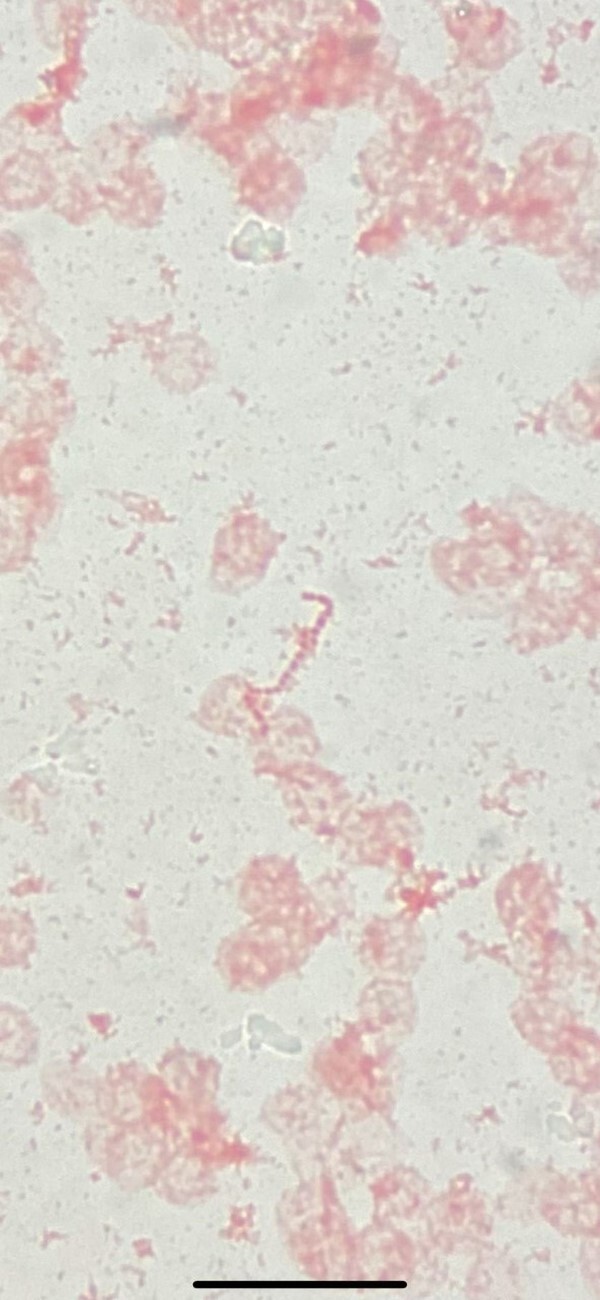
Gram stain of blood culture showing Gram-positive cocci in chains, later identified as *Streptococcus gordonii*.

An electrocardiogram showed sinus rhythm, normal PR interval, and T wave inversion in leads V2–V4. A chest radiograph showed clear lung fields. Blood tests showed normocytic anaemia with Hb 112 g/L, mean corpuscular volume of 88 fL, normal white cell count of 9.6 × 10^9^g/L, and an elevated C-reactive protein of 81 mg/L. Urine culture showed no growth. Two subsequent sets of blood cultures grew *S. gordonii*, sensitive to penicillin with a minimum inhibitory concentration of 0.012 µg/mL (*Figure 1*).

**Figure 2:**
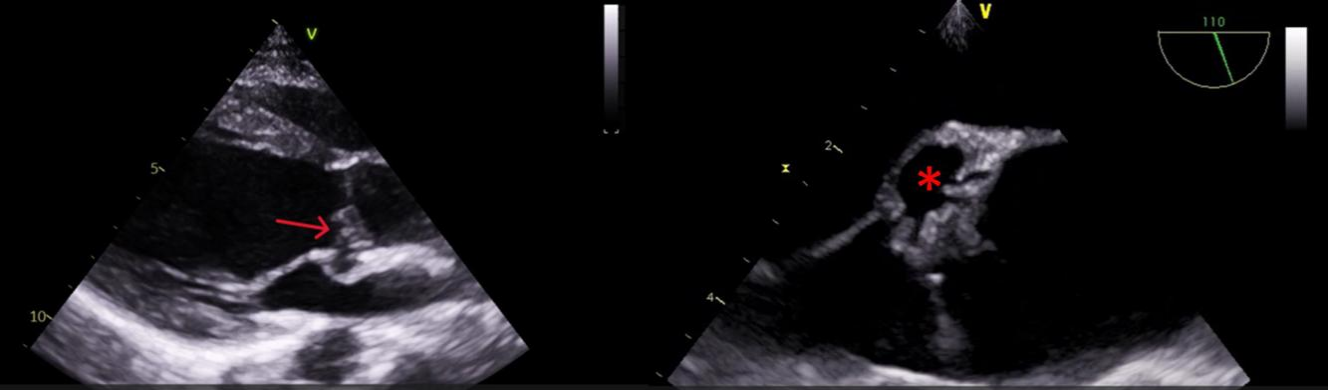
Parasternal long-axis transthoracic view of aortic valve vegetations marked by arrow (left). Mid-oesophageal long-axis view focussing on aortic valve demonstrating abscess cavity marked by asterisk (right).

A transthoracic echocardiogram showed a BAV, moderate aortic regurgitation, and possible aortic root abscess. The left ventricle was mildly dilated (left ventricular internal diameter in diastole 5.3 cm) with concentric remodelling, and the ejection fraction was 57%. A transoesophageal echocardiogram revealed aortic valve vegetations and an anechoic lesion at the aortic root not in communication with the atria. This was interpreted as an aortic root abscess (*Figure 2*). There was severe aortic regurgitation with a valvular jet directed towards the anterior mitral valve leaflet in early diastole, followed by a mid-late diastolic septally directed jet originating through the abscess cavity (see Supplementary material online, *Videos S1*–*S3*). This confirmed a diagnosis of native aortic valve infective endocarditis secondary to *S. gordonii*.

**Figure 3:**
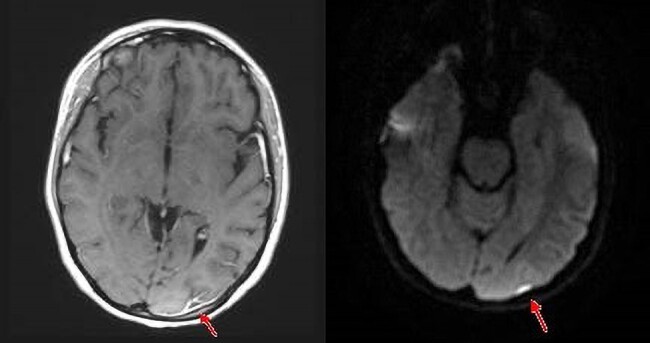
Magnetic resonance imaging brain axial T1-weighted post-contrast (left) shows a small fluid collection with an enhancing membrane overlying the left occipital lobe. Diffusion-weighted imaging (right) shows the collection returns high signal due to restricted water diffusion that is characteristic of pus.

Two days later, the patient reported headaches and visual disturbance without focal neurology. A magnetic resonance imaging brain revealed dural enhancement overlying the left occipital lobe with associated signal change reported as a small empyema (*Figure 3*).

On 14 February 2022, she was commenced on 2 g amoxicillin IV 6 hourly. Subsequent blood cultures were negative by 17 February.

The cardiothoracic surgeons offered her mechanical or bioprosthetic valve replacement. She opted for mechanical valve replacement to reduce the likelihood of requiring redo surgery. Surgery was performed 5 days after admission. Intra-operatively, vegetations were present on the BAV, and a sub-valvular cavity was closed with a pericardial patch. This native aortic valve showed no growth after prolonged incubation. She received a 19 mm On-X mechanical aortic valve and post-operatively commenced warfarin therapy.

After 2 weeks of inpatient antibiotics, she was discharged under the outpatient parenteral antibiotic therapy service on once-daily ceftriaxone for 4 weeks.

She was followed up by clinical infection and cardiothoracic surgery services. A further MRI brain on 28 April 2022 showed resolution of the cerebral empyema, and she remains well with no further neurological symptoms.

## Discussion

*Streptococcus gordonii*, a member of the VGS, is an oral commensal and opportunistic pathogen. Oral trauma breaches vascular endothelial surfaces, allowing *S. gordonii* to bind to heart valves and form biofilms. Cell wall components acting as virulence factors potentiate an inflammatory cascade, leading to infective endocarditis.^2^

Bicuspid aortic valve is the most common congenital cardiac abnormality affecting 1–2% of the general population. Abnormal valve anatomy and resultant blood flow disruption predispose to valvulopathies, aortopathies, and infective endocarditis.^3^

Data suggest patients with BAV have a 30-fold increased risk of infective endocarditis compared with the general population.^4^ This cohort may sustain similar rates of complications to traditional high-risk groups (those with previous infective endocarditis, prosthetic valves, and untreated cyanotic congenital heart disease) and have a greater likelihood of requiring cardiac surgery.

Microbiological aetiologies of BAV infective endocarditis favour VGS of odontologic origin and may not require aggressive disruption of the oral mucosa. The dental scaling procedure in this case post-dated the onset of fevers but may have contributed to bacterial load and intra-cardiac seeding.

Recommendations regarding antibiotic prophylaxis for infective endocarditis are derived from epidemiological studies, cost efficacy analyses, and antibiotic stewardship considerations. The European Society of Cardiology 2023 guidelines do not routinely recommend antibiotic prophylaxis for intermediate-risk groups such as those with BAV but rather limit its application to high-risk groups undergoing at-risk dental procedures. Oral and cutaneous hygiene should be emphasized in both cohorts.^5^

Paravalvular complications of aortic valve endocarditis include abscess or pseudoaneurysm. Pseudoaneurysms are typically located at the mitral-aortic inter-valvular fibrosa. A review of our echocardiogram images demonstrated a sub-valvular cavity at the aortic root, with no communication between the aorta and atria, typical of abscess rather than pseudoaneurysm. In challenging cases, cardiac CT or MRI provides further anatomical detail.^6^

Surgery for native aortic valve endocarditis involves either mechanical or bioprosthetic valve replacement. Whilst mechanical valves favour durability, lifelong anticoagulation is required. A patient-centred decision considering age, comorbidities, child-bearing potential, and anticoagulation adherence is paramount. Mechanical valves are conventionally avoided in those with significant intra-cranial bleeding.^7^ Peri-aortic abscess may necessitate closure of cavity defect using a patch or root reconstruction with homograft. The Ross procedure, in which a pulmonary autograft replaces the infected aortic valve, has demonstrated optimal long-term outcomes and may be an option for select younger patients in whom avoidance of anticoagulation is desirable.^8^

Neurological sequelae of infective endocarditis include septic cerebral emboli manifesting as ischaemic stroke, intra-cerebral abscess, meningitis, and intra-cerebral haemorrhage secondary to rupture of mycotic aneurysms or haemorrhagic transformation of established infarcts. The incidence of neurological sequelae is approximately 50%, including both manifest and silent events.^9^

Our patient’s MRI brain scan demonstrated an established occipital empyema that was felt to have pre-dated her presentation and perhaps non-contributary to her symptoms of headache and visual disturbance.

Due consideration must be given to proceeding with cardiac surgery in the context of neurological complications as intra-operative systemic anticoagulation for cardiopulmonary bypass surgery and disruption to cerebral perfusion homeostasis may increase the risk of intra-cerebral haemorrhage. Small lesions incidentally detected on MRI sequences have lower haemorrhagic tendencies. Accordingly, the European Society of Cardiology 2023 guidelines do not advocate for deferral of surgery in the context of transient ischaemic events or silent emboli.^5^

## Conclusion

*Streptococcus gordonii* infective endocarditis may be more frequent in patients with BAV with a dental source. Maintaining a high clinical suspicion is imperative for timely diagnosis given the significant morbidity and mortality. Early involvement of the endocarditis team through prompt initiation of antimicrobial therapy and early surgical intervention is key to improving clinical outcomes.

## Supplementary Material

ytad590_Supplementary_Data

## Data Availability

The data underlying this article are available in the article and in its online Supplementary material.

## Lead author biography

Alysha Bhatti is an internal medical trainee at St George’s Hospital, London. She graduated from Imperial College London in 2018, having completed an intercalated BSc in Clinical Pharmacology in addition to her medical studies. She is an aspiring cardiologist and is currently the Secretary of the British Junior Cardiologists’ Association (BJCA) Starter committee.

## Notes

**Consent:** The authors confirm that consent for publication of the case report has been obtained from the individual in accordance with COPE best practice guidelines.

**Funding:** None declared.
